# Genetic determinants of obesity heterogeneity in type II diabetes

**DOI:** 10.1186/s12986-020-00476-6

**Published:** 2020-07-09

**Authors:** Somayeh Alsadat Hosseini Khorami, Mohd Sokhini Abd Mutalib, Mohammad Feili Shiraz, Joseph Anthony Abdullah, Zulida Rejali, Razana Mohd Ali, Huzwah Khaza’ai

**Affiliations:** 1 Department of Nutrition and Dietetic, University Putra Malaysia, 43400 Serdang, Selangor Malaysia; 2 Department of Artificial Intelligence and Computer Engineering, Faculty of Electrical Engineering, Computer and IT, Qazvin Branch, Islamic Azad University, Qazvin, Iran; 3 Consultant Physician at Perak Community Hospital, Ipoh, Malaysia; 4 Department of Obstetrics and Gynaecology, University Putra Malaysia, 43400 Serdang, Selangor Malaysia; 5 Department of Pathology, University Putra Malaysia, 43400 Serdang, Selangor Malaysia; 6 Department of Biomedical Science, University Putra Malaysia, 43400 Serdang, Selangor Malaysia

**Keywords:** Obesity paradox, *PI3K/AKT* pathway, *PDK1*, Non-obese diabetic, Type II diabetes, Gene expression, RT-PCR

## Abstract

**Background:**

Although obesity is considered as the main cause of Type II diabetes (T2DM), non-obese individuals may still develop T2DM and obese individuals may not.

**Method:**

The mRNA expression of *PI3K/AKT* axis from 100 non-obese and obese participants with insulin sensitivity and insulin resistance states were compared in this study toward the understanding of obesity heterogeneity molecular mechanism.

**Result:**

In present study, there was no statistically significant difference in gene expression levels of *IRS1* and *PTEN* between groups, whereas *PI3K*, *AKT2* and *GLUT4* genes were expressed at a lower level in obese diabetic group compared to other groups and were statistically significant. *PDK1* gene was expressed at a higher level in non-obese diabetic group compared to obese diabetic and non-obese non-diabetics groups. No statistically significant difference was identified in gene expression pattern of *PI3K/AKT* pathway between obese non-diabetics and non-obese non-diabetics.

**Conclusion:**

The components of *PI3K/AKT* pathway which is related to the fasting state, showed reduced expression in obese diabetic group due to the chronic over-nutrition which may induced insensitivity and reduced gene expression. The pathogenesis of insulin resistance in the absence of obesity in non-obese diabetic group could be due to disturbance in another pathway related to the non-fasting state like gluconeogenesis. Therefore, the molecular mechanism of insulin signalling in non-obese diabetic individuals is different from obese diabetics which more investigations are required to study insulin signalling pathways in greater depth, in order to assess nutritional factors, contribute to insulin resistance in obese diabetic and non-obese diabetic individuals.

## Introduction

The relationship between insulin resistance and obesity is well recognised while the responsible mechanisms of obesity-related insulin resistance remain poorly understood [1–3]. Even though obesity involves enhanced risk for development of diabetes, there are some paradoxes in relationships between diabetes and obesity and no unifying hypothesis has been proposed to explain these paradoxical phenomena. It cannot be ruled out that, 15% of diabetic individuals in North America and 50% of them in Europe are non-obese (BMI < 30) diabetic. Also, more than 60% of diabetic individuals in Asia are non-obese even while the obesity criteria for Asia was adjusted from BMI > 30 to BMI > 27.5 [4]. Therefore, other factors besides or instead of obesity may contribute to development of non-obese Type II diabetes.

Recent progress in research methodology on signaling pathways has identified the insulin signal transduction and documented its alterations in insulin-resistance state [5–7]. Insulin-stimulated glucose transport occurs via *PI3K/AKT*-dependent pathway (Phosphatidylinositol 3-kinase/Protein Kinase B) which results in *GLUT4* (Glucose Transporter 4) translocation to the plasma membrane to mediate glucose uptake and activation of glycogen synthase [8]. By stimulation of insulin, *PI3K* phosphorylates membrane phospholipids and converts PIP2 (Phosphotidylinositol-4,5-bisphosphate) to PIP3 (Phosphotidylinositol-3,4,5-triphosphate). This complex phosphorylates/activates the *PDK1* and *PDK2* (Phosphoinositide-Dependent Kinase) leading to activation of *AKT/PKB* and *PKC* (Protein Kinase C) phosphorylation to translocate *GLUT4* to the plasma membrane from intracellular vesicular compartment [9]. *PI3K/AKT* pathway can be reversely dephosphorylated by *PTEN* phosphatase (Phosphatase and TENsin homolog deleted on chromosome 10) through converting PIP3 back to PIP2 [10]. All these events are related to short-term post-translational regulation of protein functions and long-term transcriptional regulation [11].

Insulin resistance in Type II diabetes has been characterized by several defects in the insulin signaling cascade [8, 12–14]. This hypothesis is supported by findings of altered expression of genes encoding metabolic pathways in Type II diabetic patients [15] such as, insulin-induced activity of *IR*, *IRS*, *PI3K*, *AKT* and *GLUT4* have been reported to be reduced in Type II diabetes [16–19]. Understanding of these alterations may explain the heterogeneity of obesity and its manifestations. The pathogenesis of insulin resistance in absence and presence of obesity is unknown and more investigations are required to study insulin signalling pathways in greater depth to assess nutritional factors contribute to insulin resistance in non-obese diabetic and obese diabetic individuals separately.

Type II diabetes is a multifaceted disease resulting from the interaction of genetics, epigenetics, lifestyle such as diet and environmental as the contributing factors. These risk factors induce or suppress expression of genes involved in insulin signaling [20]. Nutrition plays a key role in pathogenesis of diabetes and nutrient gene interactions may modulate gene expression of insulin signaling component directly or via their metabolites [21]. Blood samples collected in the framework of gene expression and epidemiological studies allow the use of humans as the model system, as opposed to using cell lines or animal models [22].

The aim of this study was to investigate the gene expression pattern of *PI3K/AKT* pathway in obese and non-obese metabolically healthy individuals and compare this pattern with obese and non-obese diabetics to propose molecular mechanistic insights into how differential regulation of *PI3K/AKT* pathway is responsible for obesity heterogeneity in Type II diabetes.

## Research design and methods

### Study protocol and participants characteristics

A total of 50 Type II diabetic and 50 non-diabetic individuals recruited in the context of a cross-sectional study on insulin signal transduction at the Universiti Putra Malaysia and Serdang Hospital. Diabetic and non-diabetic participants were divided to two groups based on category of body mass index (BMI < 30 and BMI > 30) to find out differences in molecular mechanism underlying regulation of *PI3K/AKT* pathway. In diabetic and non-diabetic group, 50% were normal/overweight (BMI < 30 kg/m2) and 50% were obese (BMI of > 30 kg/m2). This weight distribution was not significantly different between four groups. The age ranged from 35 to 60 years and there was no statistically significant difference between groups (P>0.05). Participants who had cancers, nephropathy complications, thyroid & parathyroid diseases and pregnant women were excluded from this study. Also, participants were excluded if they reported using any form of tobacco, cigarettes and alcoholic beverages. The study was approved by the ethics committee of Universiti Putra Malaysia and National Medical Research Register (NMRR & MREC). Also, the study was conducted in accordance to the Declaration of Helsinki in its currently applicable version and the guidelines of the International Conference on Harmonization of Good Clinical Practice (ICH-GCP). All participants gave written informed consent before taking part in the study.

### Study design

The mRNA expression of insulin signalling components from i) obese Type II diabetic participants (OD) and obese non-diabetics (OND), ii) non-obese Type II diabetic participants (NOD) and non-obese non-diabetics (NOND), iii) obese Type II diabetic participants (OD) and non-obese diabetics (NOD), iv) obese non-diabetic participants (OND) and non-obese non-diabetics (NOND) were compared in this study toward the understanding of obesity heterogeneity molecular mechanism.

*Assessment of clinical and metabolic characteristics of participants.* FBS (Cat.No:04657527) was tested by colorimetric enzymatic method using Roche Diagnostics GmbH Cobas-c 311 Germany machine. HbA1c analysis was performed by the colorimetric method (Diazyme; cat. No. DZ168A, USA) and C-peptide level was determined by ELISA Kit (Cloud Clone Corp; cat. no. CEA447Hu, UK).

### Gene expression analysis

Blood samples were collected after an overnight fast. Total RNA was isolated by using PAXgene Blood RNA Tubes (Qiagen; cat. no. 762165) according to the manufacturer protocol and it was converted to cDNA by using the Qiagen cDNA Synthesis Kit (Qiagen; cat. no. 205313). Real time PCR (RT-PCR) reactions were performed by using SYBR green technology and QuantiTect Rev. Transcription Kit (Qiagen; cat. no. 205313, Germany). The final volume of the RT-PCR reaction mixture was 20 μl and contained 2 μl cDNA, 1 μM of each primer, 10 μl Master Mix and RNase-free water up to 20 μl. RNA concentration and purity were measured by using a Nano Drop ND1000 spectrophotometer at A260:A280 ratio (Thermo Fisher Scientific; USA). RNA quality and integrity were determined via agarose gel electrophoresis. A positive control containing all the components of the reaction in except of template and a negative control containing template without reverse transcriptase were used to ensure quality of run and confirm the absence of contamination. RT-PCR was performed on a Bio-Rad cycler (Bio-Rad; CFX96, USA) and cycling conditions were as follows: 95 °C for 5 min (PCR initial activation step); 95 °C for 10 s (denaturation); 60 °C for 30 s (combined annealing/extension); followed by 40 cycles.

Real-time PCR results were calculated according to the ∆∆CT method by using *GAPDH* and *β-ACTIN* as housekeeping genes.

### Statistical analyses

Each experiment was performed triplicate and all data were expressed as means ± SE. Statistical analysis was performed by using SPSS 21.0 statistical software package (SPSS Inc., Chicago, IL, USA). The univariate normality assumptions were verified with the Shapiro-Wilk test. Independent-samples t-test was applied to compare the means between two groups and threshold of significance was defined as a *P* < 0.05.

## Results

Clinical and metabolic characteristic data of participants involved in this study is summarised in Table 1.

**Table 1:** Clinical and Metabolic Characteristic Data of Participants

Study Variables	OD (***n =*** 25) Mean ± SE	NOD (***n =*** 25) Mean ± SE	OND (***n =*** 25) Mean ± SE	NOND (***n =*** 25) Mean ± SE
**Age**	50.84 ± 1.54	55.28 ± 1.89	47.53 ± 1.97	45.78 ± 1.57
**Female** (%)	52	48	52	48
**BMI**	35.71 ± 1.90	28.68 ± 0.78	33.55 ± 1.01	27. ± 1.38
**FBS** (mmol/L)	8.19 ± 0.14	8.57 ± 0.17	5.51 ± 0.08	5.28 ± 0.11
**HbA1c** (%)	8.05 ± 0.14	7.79 ± 0.40	5.57 ± 0.71	5.14 ± 0.35
**C-Peptide** (ng/mL)	1.96 ± 0.03	1.97 ± 0.01	2.00 ± 0.02	2.04 ± 0.04

*OD* obese diabetic participants, *OND* obese non-diabetics, *NOD* non-obese Type II diabetic participants, *NOND* non-obese non-diabetics

Although the *PI3K/AKT* pathway is responsible for glucose uptake from blood stream to the target cells, the underlying mechanism has not been clarified yet. In current study, gene expression pattern of the components of this pathway has been identified and compared among the participants based on obesity and insulin sensitivity state, as demonstrated in Tables 2, 3, 4 and 5.

**Table 2:** Relative Expression Levels for GOIs between Obese Diabetic and Obese Non-Diabetic Participants

GOIs	OD (***n =*** 25) Mean ± SE	OND (***n =*** 25) Mean ± SE	df	t	***P***-value
***IRS1***	0.23 ± 0.01	0.26 ± 0.02	48	−0.96	0.342
***PI3K***	0.53 ± 0.03	0.32 ± 0.03	48	4.03	0.001*
***PDK1***	0.63 ± 0.05	0.68 ± 0.08	48	−1.27	0.757
***AKT2***	1.14 ± 0.08	0.65 ± 0.07	48	3.39	0.001*
***GLUT4***	1.13 ± 0.11	0.79 ± 0.08	48	2.27	0.028*
***PTEN***	0.42 ± 0.04	0.46 ± 0.04	48	−0.74	0.463

All data was expressed in percentage and mean ± standard deviation (SD). *Significant level is at 0.05 *p*-value

**Table 3:** Relative Expression Levels for GOIs between Non-Obese Diabetic and Non-Obese Non-Diabetic Participants

GOIs	NOD (***n =*** 25) Mean ± SE	NOND (***n =*** 25) Mean ± SE	df	t	***P***-value
***IRS1***	0.20 ± 0.02	0.20 ± 0.02	48	−0.28	0.774
***PI3K***	0.38 ± 0.03	0.32 ± 0.03	48	1.26	0.212
***PDK1***	0.51 ± 0.04	0.70 ± 0.07	48	−2.40	0.001*
***AKT2***	0.57 ± 0.05	0.70 ± 0.06	48	−1.59	0.115
***GLUT4***	0.79 ± 0.08	0.69 ± 0.07	48	0.79	0.432
***PTEN***	0.43 ± 0.04	0.43 ± 0.05	48	0.02	0.982

All data was expressed in percentage and mean ± standard deviation (SD). *Significant level is at 0.05 *p*-value

**Table 4:** Relative Expression Levels for GOIs between Obese Diabetic and Non-Obese Diabetic Participants

GOIs	NOD (***n*** = 25) Mean ± SE	OD (***n =*** 25) Mean ± SE	df	t	***P-***value
***IRS1***	0.20 ± 0.02	0.23 ± 0.01	48	−1.04	0.299
***PI3K***	0.38 ± 0.03	0.53 ± 0.03	48	−2.97	0.005*
***PDK1***	0.51 ± 0.02	0.63 ± 0.05	48	−4.93	0.001*
***AKT2***	0.57 ± 0.05	1.14 ± 0.09	48	−4.59	0.001*
***GLUT4***	0.79 ± 0.08	1.13 ± 0.11	48	−2.33	0.024*
***PTEN***	0.43 ± 0.04	0.42 ± 0.04	48	0.26	0.792

All data was expressed in percentage and mean ± standard deviation (SD). *Significant level is at 0.05 *p*-value

**Table 5:** Relative Expression Levels for GOIs between Obese Non-Diabetic and Non-Obese Non-Diabetics Participants

GOIs	NOND (***n*** = 25) Mean ± SE	OND (***n =*** 25) Mean ± SE	df	t	***P-***value
***IRS1***	0.20 ± 0.02	0.26 ± 0.02	48	−1.54	0.130
***PI3K***	0.32 ± 0.03	0.32 ± 0.03	48	−0.10	0.914
***PDK1***	0.70 ± 0.07	0.68 ± 0.08	48	−0.13	0.897
***AKT2***	0.70 ± 0.06	0.65 ± 0.06	48	0.45	0.654
***GLUT4***	0.69 ± 0.07	0.79 ± 0.08	48	−0.85	0.395
***PTEN***	0.43 ± 0.04	0.46 ± 0.05	48	−0.42	0.674

All data was expressed in percentage and mean ± standard deviation (SD). Significant level is at *p-*value **< 0.05.**

*PI3K*, *AKT2* and *GLUT4* expression levels were significantly lower in obese diabetic participants compared to obese non-diabetics. *PDK1* expression level was significantly higher in non-obese diabetic participants compared to non-obese non-diabetics. The obese diabetic and non-obese diabetic groups were significantly different based on *PI3K*, *AKT2*, *GLUT4* and *PDK1* gene expression levels. The gene expression levels of *PI3K*, *AKT2* and *GLUT4* were significantly lower in obese diabetic participants compared to non-obese diabetics whereas the gene expression level of *PDK1* was significantly higher in non-obese diabetics compared to obese diabetics. No statistically significant gene expression difference was identified between non-obese non-diabetics and obese non-diabetics.

These results provide evidence that non-obese diabetics might be different from obese diabetics, also obese diabetics might be different from obese non-diabetics in mechanism of insulin signal transduction which causes obesity heterogeneity.

Expression levels of *PI3K*, *AKT2* and *GLUT4* were significantly lower in obese Type II diabetic participants in comparison to the obese non-diabetics.

There was no significant difference in gene expression pattern of *PI3K/AKT* pathway between non-obese diabetic and non-obese non-diabetics in except of higher expression level of *PDK1* in non-obese diabetics.

The gene expression levels of *PI3K*, *AKT2* and *GLUT4* were significantly lower in obese diabetic participants compared to non-obese diabetics whereas the gene expression level of *PDK1* was significantly higher in non-obese diabetics.

The non-obese non-diabetic and obese non-diabetic groups were not significantly different based on *PI3K/AKT* pathway genes.

Likewise, there was no difference in the trends of the data when men and women were analysed separately.

## Discussion

The main strength of this study is the inclusion of non-obese and obese participants with insulin sensitivity and insulin resistance states. This study design allowed for a simultaneous investigation on the influence of obesity and diabetes on *PI3K/AKT* pathway toward the identification of genetic determinants of obesity heterogeneity in Type II diabetes. Consideration of only *PI3K/AKT* axis of insulin signalling pathways limited interpretations to this study.

Even though other biochemical and physiological differences may exist between OD and NOD group beyond their BMI, the results of this study suggest that the gene expression pattern of *PI3K/AKT* pathway in NOD group is more closely resemble to the gene expression pattern of OND and NOND groups and differs from OD group. Indeed, significant higher gene expression level of *PDK1* in NOD group compared to other groups emphasizes disturbance in another signaling pathway in NOD individuals.

Therefore, it can be hypothesized that insulin resistance and obesity might be distinct risk factors for metabolic disturbances [23]. Furthermore, it has been revealed that the presence of obesity does not necessarily cause alterations in *PI3K/AKT* pathway gene expression and insulin resistance and Type II diabetes is not necessarily associated with obesity and alterations in *PI3K/AKT* pathway gene expression. However, this study has revealed that disturbed *PI3K/AKT* pathway genes expression was associated with obesity and insulin resistance. Classification of obesity into metabolically healthy obesity and metabolically unhealthy obesity can also be explained by this hypothesis. Insulin resistance occurs when obesity is accompanied by reduced gene expression of *PI3K/AKT2/GLUT4* pathway. An obese individual with a normal *PI3K/AKT2/GLUT4* gene expression level would be still metabolically healthy until the time body is not able to keep this ability. Another assumption is that their adipocytes are able to switch from glucose to fatty acid metabolism which may be beneficial in combating metabolic syndrome [24] or the combination of their diet is different from OD group which further details will be discussed. Also, it has been reported that there is no significant differences in BMI, waist circumferences and fat mass between European NOD and control individuals [25], hence, visceral fat is not associated with non-obese diabetes. Since, insulin resistance is not associated with obesity and alterations in *PI3K/AKT* pathway in NOD individuals, disturbances in other insulin signalling pathways are proposed.

Two major downstream pathways of insulin action are the *PI3K/AKT* (phosphatidylinositol 3-kinase/protein kinase B) and *MAPK* (mitogen-activated protein kinases) pathways [26, 27]. In this study the *PI3K/AKT* pathway has been considered which is related to the fasting state, hence the components of this pathway showed alterations in OD group as a result of over nutrition. Over-stimulated *PI3K/AKT* pathway due to the chronic over-nutrition may induced insensitivity. This “overstimulation-induced insensitivity” phenomenon is commonly present in almost all of the metabolic disorders [28] which may cause reduced gene expression level in *PI3K/AKT* pathway in OD group. Therefore, pathogenesis of insulin resistance in the absence of obesity in NOD individuals could be related to other pathways of insulin signaling [29, 30].

The findings of present study showed reduced expression level of *PI3K, AKT2* and *GLUT4* in OD group compared to other groups. Nevertheless, there was no significant difference in gene expression level of *PTEN* and *IRS1* in OD, NOD, OND and NOND groups.

No significant difference in *IRS1* expression level might be due to the collaboration of various mechanisms including signal amplification as a compensatory mechanism and convergence of other signalling pathways. However, the role of negative feedback loops cannot be neglected, as control of insulin signalling can be achieved by autoregulation whereby downstream elements inhibit upstream components [31, 32].

It has been documented that overexpression of *PTEN* decreases insulin-stimulated *PI3K/AKT* pathway, *GLUT4* translocation and glucose uptake into the cells [18, 33]. Microinjection of anti-*PTEN* antibody increases insulin-stimulated *GLUT4* translocation to the cell membrane and glucose uptake [33]. No significant difference in *PTEN* expression level might be related to the collaboration or interaction of *PTEN* with other phosphatases in antagonizing *PI3K/AKT* pathway and induction of diabetes. Therefore, further studies are required to differentiate the roles of different phosphatases, their interactions and collaborations. Since the main role of *PTEN* is performed by dephosphorylating the stimulated form of insulin receptor and modulating post-receptor signalling through converting PIP3 back into PIP2 and reversing the effects of *PI3K/AKT* pathway [34–36], its transmembrane function might be more imperative than its intracellular function [37–39].

Although *MAPK* insulin signalling pathway was not investigated in this study, previous data from researchers [40] support the idea that alterations in *MAPK* signal transduction contributes to development of Type II diabetes as well. The coordinated regulation of glucose uptake from circulation and endogenous glucose production is indispensable to maintain constant blood glucose level. The liver contributes to this process through controlling the gluconeogenesis and glycogenolysis. Suppression of endogenous glucose production during the postprandial state is attributed to insulin. Moreover, insulin activates glycogen synthase while simultaneously inactivates phosphorylase a and phosphorylase kinase to control glycogen metabolism through the *MAPK* pathway [27]. Mice lacking *MAPK* phosphatase-1 exhibited hepatic insulin resistance and increased gluconeogenesis [41]. Thus, transcriptional regulation of key gluconeogenic genes (Glucose-6-phosphatase, Pyruvate carboxylase and PEP carboxykinase) is as important as glucose metabolism genes (*PI3K/AKT* pathway components). Transcription factor *FOX01*, has been identified as a main regulator of gluconeogenesis [42]. In the fed state, insulin signaling activates *PI3K/AKT* axis and subsequently phosphorylates/inactivates *FOX01* to inhibit gluconeogenesis [43]. Hyperglycemia following hepatic deletion of *AKT* could be corrected by concomitant hepatic deletion of *FOX01* [43, 44]. Specific deletion of *FOX01* in the liver of mice caused reduced gluconeogenic genes expression level and fasting glucose. Also, glucose clamp studies demonstrated that *FOX01* ablation impairs fasting gluconeogenesis and glycogenolysis.

**Fig. 1:**
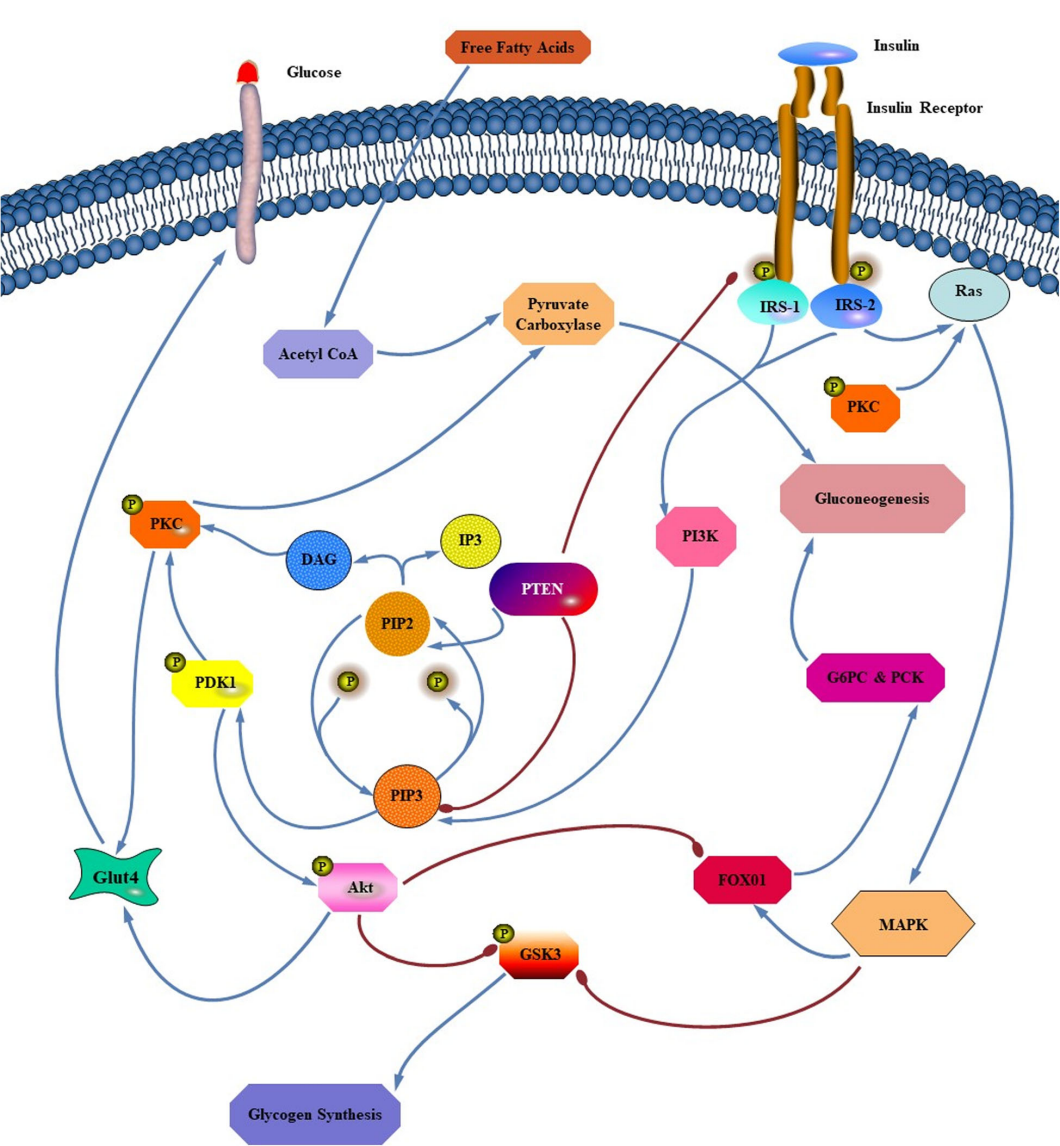
Coordinated regulation of exogenous glucose uptake from circulation and endogenous glucose production through gluconeogenesis and glycogenolysis. Insulin receptors stimulate phosphorylation/activation of *IRS* and subsequently activation of *PI3K* cascade which is the main pathway involved in glucose transport. *PI3K* stimulates phosphorylation of *AKT* and *GSK3*, which increases and decreases their activities respectively. The function of the previously mentioned kinases of *PI3K/AKT* pathway is antagonized by *PTEN* phosphatase. *AKT* phosphorylates/inactivates *GSK3* and increases glycogen synthase activity. Also, *AKT* inhibits *FOX01* which results in inhibition of gluconeogenesis by suppressing *G6pc* and *Pck1*. *PI3K* via *PDK1* activates *PKC* that stimulates gluconeogenesis and contributes to insulin resistance by converting free fatty acids to Acetyl-CoA, which activates pyruvate carboxylase

It has been known that *PKC* is an activator of gluconeogenesis through activation of *MAPK* pathway [45, 46] besides stimulation of pyruvate carboxylase [47]. Activation of *MAPK* pathway stimulates *FOX01* axis to increase Glucose-6-phosphatase and PEP carboxykinase. *PI3K* via *PDK1* activates *PKC*, which stimulates gluconeogenesis and contributes to insulin resistance by converting free fatty acids to Acetyl-CoA, which activates pyruvate carboxylase [47] (Fig. 1).

Consequently, the insulin regulation of endogenous and exogenous glucose metabolism and maintenance of glucose homeostasis are mediated though *PI3K/AKT* and *MAPK* pathways [43]. In this case, the involvement of nutritional factors may pronounce in stimulation or inhibition of different insulin signalling pathways. A selective model of insulin resistance can be proposed to explain the paradoxical findings related to the obesity and Type II diabetes. In non-obesity insulin resistance state, there might be a selective loss of insulin function possibly through *PKC* axis instead of *AKT* (*PKB*) axis. Though, this study did not directly address the effect of *PKC* activation on selective insulin resistance, contribution of endogenous glucose production is proposed in NOD individuals as a result of increased gene expression level of *PDK1 *in this group which is the main regulator of *PKC* axis. This selective model of insulin resistance might be induced by different nutritional stimulators and a combination of nutritional and hormonal model that includes multiple modes of insulin signalling pathways regulations is proposed [47]. This model can explain experimental findings and demonstrates how several feedback loops based on different nutritional stimulators can produce obesity heterogeneity. Determining nutritional mediators which stimulate different molecular mechanisms of insulin signalling to generate these paradoxical effects provides insights into the obesity heterogeneity in Type II diabetes.

It has been suggested that ketogenic diets and high glycemic index or glycemic load diets develop Type II diabetes through endogenous glucose production and emphasizes the role of nutritional induction for obesity paradox in diabetes.

ketogenic diets induce severe hepatic insulin resistance in mice through impairments in insulin suppression of endogenous glucose production during a hyper-insulinemic euglycemic clamp. These researchers claimed that hepatic insulin resistance could be attributed to the increase of hepatic triglycerides and diacylglycerol content which may cause reduced insulin signalling through activation of *PKC* [48, 49]. Mice lacking *PKC* were protected from diet-induced insulin resistance, even with increased hepatic lipid content [50]. Also, gene expression of *FOX01* is increased in non-alcoholic steatohepatitis individuals that emphasizes the role of disturbed lipid metabolism in dysregulation of gluconeogenesis [51]. Contradictory findings in research of hepatic metabolism indicate distinct adaptive metabolism in starvation, ketogenic diets and fasting states.

It has been suggested that chronic exposure to high glycemic index and high glycemic load diets stimulate more production of insulin, resulting in hyper-insulinemia and insulin resistance due to the pancreas exhaustion and failure [52]. Moreover, high glycemic index and/or high glycemic load diets increase blood glucose and free fatty acids [53]. Elevated blood glucose can induce higher peak of insulin and low post-prandial glucose which may stimulate glycogenolysis and gluconeogenesis [53]. Elevated free fatty acids may stimulate gluconeogenesis through activation of *PKC* axis [47].

Another proof of induced insulin resistance through endogenous glucose production is hyperglycemia induced by psychiatric stress that play role in pathogenesis of Type II diabetes [54]. The level of hyperglycemia induced by psychiatric stress is difficult to elucidate as it is impossible to produce and/or quantify in humans due to the ethical and methodological considerations. Glucagon, epinephrine and cortisol as stress hormones cause hyperglycemia through stimulation of gluconeogenesis and glycogenolysis in liver, inhibition of insulin secretion and interruption of glucose absorption in muscle [55–57]. It has been documented that epinephrine stimulates glucagon to produce glucose in stress situation, whereas cortisol maintains elevated glucose by epinephrine and glucagon in post-stress phase [58, 59].

Another molecular mechanism associated with insulin receptor regulation which is not considered in this study is *MAPK* pathway that controls metabolism and contributes in regulation of gluconeogenesis. Therefore, the pathogenesis of insulin resistance in the absence of obesity in NOD individuals could be due to the other pathways related to the non-fasting states like gluconeogenesis. Indeed, the contribution of endogenous glucose production may have been underestimated in NOD individuals.

## Conclusion

Evidently, diabetes and obesity are a complex disease with a large pool of genes that are involved in its development. It can be postulated that obesity is an independent or distinct risk factor of Type II diabetes while the impaired gene expression of different insulin signalling pathways would be the main risk factors which some of these disorders may accompany by obesity and some may not. This hypothesis offers an effective explanation for the noted paradoxes of diabetes and obesity.

In this study the *PI3K/AKT* pathway has been considered which is related to the fasting state, and its components showed reduced expression in obese diabetic group due to the chronic over-nutrition which may induce insensitivity and reduced gene expression level.

Although evidence of differential regulation of insulin signalling pathway based on nutritional stimulations is available, molecular mechanisms responsible for insulin resistance and obesity heterogeneity are largely speculative at this time. Future investigations are undertaken to address the question that whether nutritional mediators stimulate different molecular mechanisms of insulin signalling or endogenous glucose production is responsible for insulin resistance and these paradoxical effects for devising treatments.

## Data Availability

The data that support the findings of this study are available.

## Abbreviations

*AKT*: Protein Kinase B
*FOX01*: Transcription factor
*GLUT4*: Glucose Transporter 4
*IR*: Insulin receptor
*IRS*: Insulin-receptor substrate
*MAPK*: Mitogen-activated protein kinases
NOD: Non-obese Type II diabetic
NOND: Non-obese non-diabetic
OD: Obese Type II diabetic
OND: Obese non-diabetic
*PDK*: Phosphoinositide-Dependent Kinase
*PI3K*: Phosphatidylinositol 3-kinase
*PIP2*: Phosphotidylinositol-4,5-bisphosphate
*PIP3*: Phosphotidylinositol-3,4,5-triphosphate
*PKC*: Protein Kinase C
*PTEN*: Phosphatase and TENsin homolog deleted on chromosome 10
